# Ultrasensitive ctDNA detection for preoperative disease stratification in early-stage lung adenocarcinoma

**DOI:** 10.1038/s41591-024-03216-y

**Published:** 2025-01-13

**Authors:** James R. M. Black, Gabor Bartha, Charles W. Abbott, Sean M. Boyle, Takahiro Karasaki, Bailiang Li, Rui Chen, Jason Harris, Selvaraju Veeriah, Martina Colopi, Maise Al Bakir, Wing Kin Liu, John Lyle, Fábio C. P. Navarro, Josette Northcott, Rachel Marty Pyke, Mark S. Hill, Kerstin Thol, Ariana Huebner, Chris Bailey, Emma C. Colliver, Carlos Martínez-Ruiz, Kristiana Grigoriadis, Piotr Pawlik, David A. Moore, Daniele Marinelli, Oliver G. Shutkever, Cian Murphy, Monica Sivakumar, TRACERx consortium, James R. M. Black, Takahiro Karasaki, Selvaraju Veeriah, Maise Al Bakir, Wing Kin Liu, Mark S. Hill, Kerstin Thol, Ariana Huebner, Chris Bailey, Emma C. Colliver, Carlos Martínez-Ruiz, Kristiana Grigoriadis, Piotr Pawlik, David A. Moore, Monica Sivakumar, Jason F. Lester, Amrita Bajaj, Apostolos Nakas, Azmina Sodha-Ramdeen, Mohamad Tufail, Molly Scotland, Rebecca Boyles, Sridhar Rathinam, Claire Wilson, Domenic Marrone, Sean Dulloo, Dean A. Fennell, Gurdeep Matharu, Ekaterini Boleti, Heather Cheyne, Mohammed Khalil, Shirley Richardson, Tracey Cruickshank, Gillian Price, Keith M. Kerr, Sarah Benafif, Jack French, Kayleigh Gilbert, Babu Naidu, Akshay J. Patel, Aya Osman, Carol Enstone, Gerald Langman, Helen Shackleford, Madava Djearaman, Salma Kadiri, Gary Middleton, Angela Leek, Jack Davies Hodgkinson, Nicola Totton, Angeles Montero, Elaine Smith, Eustace Fontaine, Felice Granato, Antonio Paiva-Correia, Juliette Novasio, Kendadai Rammohan, Leena Joseph, Paul Bishop, Rajesh Shah, Stuart Moss, Vijay Joshi, Philip A. J. Crosbie, Katherine D. Brown, Mathew Carter, Anshuman Chaturvedi, Pedro Oliveira, Colin R. Lindsay, Fiona H. Blackhall, Matthew G. Krebs, Yvonne Summers, Alexandra Clipson, Jonathan Tugwood, Alastair Kerr, Dominic G. Rothwell, Caroline Dive, Hugo JWL Aerts, Roland F. Schwarz, Tom L. Kaufmann, Gareth A. Wilson, Rachel Rosenthal, Peter Van Loo, Nicolai J. Birkbak, Zoltan Szallasi, Judit Kisistok, Mateo Sokac, Roberto Salgado, Miklos Diossy, Jonas Demeulemeester, Abigail Bunkum, Angela Dwornik, Alastair Magness, Andrew J. Rowan, Angeliki Karamani, Antonia Toncheva, Benny Chain, Carla Castignani, Christopher Abbosh, Clare Puttick, Clare E. Weeden, Claudia Lee, Corentin Richard, Crispin T. Hiley, Cristina Naceur-Lombardelli, David R. Pearce, Despoina Karagianni, Dhruva Biswas, Dina Levi, Elizabeth Larose Cadieux, Emilia L. Lim, Emma Nye, Eva Grönroos, Felip Gálvez-Cancino, Francisco Gimeno-Valiente, George Kassiotis, Georgia Stavrou, Gerasimos-Theodoros Mastrokalos, Helen L. Lowe, Ignacio Garcia Matos, Imran Noorani, Jacki Goldman, James L. Reading, Jayant K. Rane, Jerome Nicod, John A. Hartley, Karl S. Peggs, Katey S. S. Enfield, Kayalvizhi Selvaraju, Kevin Litchfield, Kevin W. Ng, Kezhong Chen, Krijn Dijkstra, Krupa Thakkar, Leah Ensell, Mansi Shah, Maria Litovchenko, Mariana Werner Sunderland, Matthew R. Huska, Michelle Dietzen, Michelle M. Leung, Mickael Escudero, Mihaela Angelova, Miljana Tanić, Nnennaya Kanu, Olga Chervova, Olivia Lucas, Oriol Pich, Othman Al-Sawaf, Paulina Prymas, Philip Hobson, Richard Kevin Stone, Robert Bentham, Robert E. Hynds, Roberto Vendramin, Sadegh Saghafinia, Samuel Gamble, Seng Kuong Anakin Ung, Sergio A. Quezada, Sharon Vanloo, Simone Zaccaria, Sonya Hessey, Sophia Ward, Sian Harries, Stefan Boeing, Stephan Beck, Supreet Kaur Bola, Tamara Denner, Teresa Marafioti, Thomas B. K. Watkins, Thomas Patrick Jones, Victoria Spanswick, Vittorio Barbè, Wei-Ting Lu, William Hill, Yin Wu, Yutaka Naito, Zoe Ramsden, Catarina Veiga, Gary Royle, Charles-Antoine Collins-Fekete, Francesco Fraioli, Paul Ashford, Martin D. Forster, Siow Ming Lee, Elaine Borg, Mary Falzon, Dionysis Papadatos-Pastos, James Wilson, Tanya Ahmad, Alexander James Procter, Asia Ahmed, Magali N. Taylor, Arjun Nair, David Lawrence, Davide Patrini, Neal Navani, Ricky M. Thakrar, Sam M. Janes, Emilie Martinoni Hoogenboom, Fleur Monk, James W. Holding, Junaid Choudhary, Kunal Bhakhri, Marco Scarci, Pat Gorman, Reena Khiroya, Robert CM Stephens, Yien Ning Sophia Wong, Zoltan Kaplar, Steve Bandula, Anne-Marie Hacker, Abigail Sharp, Sean Smith, Harjot Kaur Dhanda, Camilla Pilotti, Rachel Leslie, Anca Grapa, Hanyun Zhang, Khalid AbdulJabbar, Xiaoxi Pan, Yinyin Yuan, David Chuter, Mairead MacKenzie, Serena Chee, Aiman Alzetani, Judith Cave, Jennifer Richards, Eric Lim, Paulo De Sousa, Simon Jordan, Alexandra Rice, Hilgardt Raubenheimer, Harshil Bhayani, Lyn Ambrose, Anand Devaraj, Hema Chavan, Sofina Begum, Silviu I. Buderi, Daniel Kaniu, Mpho Malima, Sarah Booth, Andrew G. Nicholson, Nadia Fernandes, Pratibha Shah, Chiara Proli, Madeleine Hewish, Sarah Danson, Michael J. Shackcloth, Lily Robinson, Peter Russell, Kevin G. Blyth, Andrew Kidd, Craig Dick, John Le Quesne, Alan Kirk, Mo Asif, Rocco Bilancia, Nikos Kostoulas, Mathew Thomas, Jacqui A. Shaw, Allan Hackshaw, Nicholas McGranahan, Mariam Jamal-Hanjani, Alexander M. Frankell, Charles Swanton, Jacqui A. Shaw, Allan Hackshaw, Nicholas McGranahan, Mariam Jamal-Hanjani, Alexander M. Frankell, Richard O. Chen, Charles Swanton

**Affiliations:** 1 Cancer Research UK Lung Cancer Centre of Excellence, University College London Cancer Institute, London, UK; 2 Cancer Evolution and Genome Instability Laboratory., The Francis Crick Institute, London, UK; 3 Personalis Inc., Fremont, CA USA; 4 Cancer Metastasis Laboratory, University College London Cancer Institute, London, UK; 5 Department of Thoracic Surgery, Respiratory Center, Toranomon Hospital, Tokyo, Japan; 6 Cancer Genome Evolution Research Group, Cancer Research UK Lung Cancer Centre of Excellence, University College London Cancer Institute, London, UK; 7 Department of Cellular Pathology, University College London Hospitals, London, UK; 8 Department of Experimental Medicine, Sapienza University, Rome, Italy; 9 Leicester NIHR BRC & University of Leicester, Leicester, UK; 10 Cancer Research UK & UCL Cancer Trials Centre, London, UK; 11 Department of Oncology, University College London Hospitals, London, UK; 12 Singleton Hospital, Swansea Bay University Health Board, Swansea, UK; 13 University Hospitals of Leicester NHS Trust, Leicester, UK; 14 Leicester Medical School, University of Leicester, Leicester, UK; 15 University of Leicester, Leicester, UK; 16 Cancer Research Centre, University of Leicester, Leicester, UK; 17 Royal Free London NHS Foundation Trust, London, UK; 18 Aberdeen Royal Infirmary NHS Grampian, Aberdeen, UK; 19 Department of Medical Oncology, Aberdeen Royal Infirmary NHS Grampian, Aberdeen, UK; 20 University of Aberdeen, Aberdeen, UK; 21 Department of Pathology, Aberdeen Royal Infirmary NHS Grampian, Aberdeen, UK; 22 The Whittington Hospital NHS Trust, London, UK; 23 Birmingham Acute Care Research Group, Institute of Inflammation and Ageing, University of Birmingham, Birmingham, UK; 24 Guy’s and St Thomas’ NHS Foundation Trust, London, UK; 25 University Hospital Birmingham NHS Foundation Trust, Birmingham, UK; 26 Institute of Immunology and Immunotherapy, University of Birmingham, Birmingham, UK; 27 Manchester Cancer Research Centre Biobank, Manchester, UK; 28 Wythenshawe Hospital, Manchester University NHS Foundation Trust, Wythenshawe, UK; 29 Manchester University NHS Foundation Trust, Manchester, UK; 30 Division of Infection, Immunity and Respiratory Medicine, University of Manchester, Manchester, UK; 31 Cancer Research UK Lung Cancer Centre of Excellence, University of Manchester, Manchester, UK; 32 The Christie NHS Foundation Trust, Manchester, UK; 33 Division of Cancer Sciences, The University of Manchester and The Christie NHS Foundation Trust, Manchester, UK; 34 Cancer Research UK Manchester Institute Cancer Biomarker Centre, University of Manchester, Manchester, UK; 35 Artificial Intelligence in Medicine (AIM) Program, Mass General Brigham, Harvard Medical School, Boston, MA USA; 36 Department of Radiation Oncology, Brigham and Women’s Hospital, Dana-Farber Cancer Institute, Harvard Medical School, Boston, MA USA; 37 Radiology and Nuclear Medicine, CARIM & GROW, Maastricht University, Maastricht, The Netherlands; 38 Institute for Computational Cancer Biology, Center for Integrated Oncology (CIO), Cancer Research Center Cologne Essen (CCCE), Faculty of Medicine and University Hospital Cologne, University of Cologne, Cologne, Germany; 39 Berlin Institute for the Foundations of Learning and Data (BIFOLD), Berlin, Germany; 40 Berlin Institute for Medical Systems Biology, Max Delbrück Center for Molecular Medicine in the Helmholtz Association (MDC), Berlin, Germany; 41 Department of Genetics, The University of Texas MD Anderson Cancer Center, Houston, Texas USA; 42 Department of Genomic Medicine, The University of Texas MD Anderson Cancer Center, Houston, Texas USA; 43 Cancer Genomics Laboratory, The Francis Crick Institute, London, UK; 44 Department of Molecular Medicine, Aarhus University Hospital, Aarhus, Denmark; 45 Department of Clinical Medicine, Aarhus University, Aarhus, Denmark; 46 Bioinformatics Research Centre, Aarhus University, Aarhus, Denmark; 47 Danish Cancer Society Research Center, Copenhagen, Denmark; 48 Computational Health Informatics Program, Boston Children’s Hospital, Boston, MA USA; 49 Department of Bioinformatics, Semmelweis University, Budapest, Hungary; 50 Department of Pathology, ZAS Hospitals, Antwerp, Belgium; 51 Division of Research, Peter MacCallum Cancer Centre, Melbourne, Australia; 52 Department of Physics of Complex Systems, ELTE Eötvös Loránd University, Budapest, Hungary; 53 Integrative Cancer Genomics Laboratory, VIB Center for Cancer Biology, Leuven, Belgium; 54 VIB Center for AI & Computational Biology, Leuven, Belgium; 55 Department of Oncology, KU Leuven, Leuven, Belgium; 56 Computational Cancer Genomics Research Group, University College London Cancer Institute, London, UK; 57 University College London Cancer Institute, London, UK; 58 The Francis Crick Institute, London, UK; 59 Medical Genomics, University College London Cancer Institute, London, UK; 60 Bill Lyons Informatics Centre, University College London Cancer Institute, London, UK; 61 Experimental Histopathology, The Francis Crick Institute, London, UK; 62 Department of Infectious Disease, Faculty of Medicine, Imperial College London, London, UK; 63 Advanced Sequencing Facility, The Francis Crick Institute, London, UK; 64 Department of Haematology, University College London Hospitals, London, UK; 65 Cancer Immunology Unit, Research Department of Haematology, University College London Cancer Institute, London, UK; 66 Tumour Immunogenomics and Immunosurveillance Laboratory, University College London Cancer Institute, London, UK; 67 Retroviral Immunology Group, The Francis Crick Institute, London, UK; 68 Bioinformatics and Systems Biology, Method Development and Research Infrastructure, Robert Koch Institute, Berlin, Germany; 69 Experimental Oncology, Institute for Oncology and Radiology of Serbia, Belgrade, Serbia; 70 University College London Department of Epidemiology and Health Care, London, UK; 71 University College London Hospitals, London, UK; 72 Department I of Internal Medicine, University Hospital of Cologne, Cologne, Germany; 73 Immune Regulation and Tumour Immunotherapy Group, Cancer Immunology Unit, Research Department of Haematology, University College London Cancer Institute, London, UK; 74 Cancer Evolution and Genome Instability Laboratory, The Francis Crick Institute, London, UK; 75 Centre for Medical Image Computing, Department of Medical Physics and Biomedical Engineering, University College London, London, UK; 76 Department of Medical Physics and Bioengineering, University College London Cancer Institute, London, UK; 77 Department of Medical Physics and Biomedical Engineering, University College London, London, UK; 78 Institute of Nuclear Medicine, Division of Medicine, University College London, London, UK; 79 Institute of Structural and Molecular Biology, University College London, London, UK; 80 Department of Radiology, University College London Hospitals, London, UK; 81 UCL Respiratory, Department of Medicine, University College London, London, UK; 82 Department of Thoracic Surgery, University College London Hospital NHS Trust, London, UK; 83 Lungs for Living Research Centre, UCL Respiratory, University College London, London, UK; 84 Department of Thoracic Medicine, University College London Hospitals, London, UK; 85 Lungs for Living Research Centre, UCL Respiratory, Department of Medicine, University College London, London, UK; 86 Integrated Radiology Department, North-buda St. John’s Central Hospital, Budapest, Hungary; 87 Institute of Nuclear Medicine, University College London Hospitals, London, UK; 88 The Institute of Cancer Research, London, UK; 89 Case45, London, UK; 90 The University of Texas MD Anderson Cancer Center, Houston, USA; 91 Independent Cancer Patients’ Voice, London, UK; 92 University Hospital Southampton NHS Foundation Trust, Southampton, UK; 93 Department of Oncology, University Hospital Southampton NHS Foundation Trust, Southampton, UK; 94 Academic Division of Thoracic Surgery, Imperial College London, London, UK; 95 Royal Brompton and Harefield Hospitals, part of Guy’s and St Thomas’ NHS Foundation Trust, London, UK; 96 National Heart and Lung Institute, Imperial College, London, UK; 97 Royal Surrey Hospital, Royal Surrey Hospitals NHS Foundation Trust, Guildford, UK; 98 University of Surrey, Guildford, UK; 99 University of Sheffield, Sheffield, UK; 100 Sheffield Teaching Hospitals NHS Foundation Trust, Sheffield, UK; 101 Liverpool Heart and Chest Hospital, Liverpool, UK; 102 Princess Alexandra Hospital, The Princess Alexandra Hospital NHS Trust, Harlow, UK; 103 School of Cancer Sciences, University of Glasgow, Glasgow, UK; 104 Beatson Institute for Cancer Research, University of Glasgow, Glasgow, UK; 105 Queen Elizabeth University Hospital, Glasgow, UK; 106 Institute of Infection, Immunity & Inflammation, University of Glasgow, Glasgow, UK; 107 NHS Greater Glasgow and Clyde, Glasgow, UK; 108 Cancer Research UK Scotland Institute, Glasgow, UK; 109 Institute of Cancer Sciences, University of Glasgow, Glasgow, UK; 110 NHS Greater Glasgow and Clyde Pathology Department, Queen Elizabeth University Hospital, Glasgow, UK; 111 Golden Jubilee National Hospital, Clydebank, UK

**Keywords:** Non-small-cell lung cancer, Tumour biomarkers

## Abstract

Circulating tumor DNA (ctDNA) detection can predict clinical risk in early-stage tumors. However, clinical applications are constrained by the sensitivity of clinically validated ctDNA detection approaches. NeXT Personal is a whole-genome-based, tumor-informed platform that has been analytically validated for ultrasensitive ctDNA detection at 1–3 ppm of ctDNA with 99.9% specificity. Through an analysis of 171 patients with early-stage lung cancer from the TRACERx study, we detected ctDNA pre-operatively within 81% of patients with lung adenocarcinoma (LUAD), including 53% of those with pathological TNM (pTNM) stage I disease. ctDNA predicted worse clinical outcome, and patients with LUAD with <80 ppm preoperative ctDNA levels (the 95% limit of detection of a ctDNA detection approach previously published in TRACERx) experienced reduced overall survival compared with ctDNA-negative patients with LUAD. Although prospective studies are needed to confirm the clinical utility of the assay, these data show that our approach has the potential to improve disease stratification in early-stage LUADs.

## Abstract (web summary)

In the TRACERx cohort of 171 patients with lung cancer, the ultrasensitive detection of ctDNA improves preoperative patient stratification, also in early-stage disease.

## Main

Liquid biopsy for detecting circulating tumor DNA (ctDNA, namely cell-free DNA derived from a tumor) holds promise as a strategy for personalized clinical management of early-stage cancers^1–7^. Preoperative ctDNA status has shown potential as a biomarker, while postoperative ctDNA detection can direct adjuvant therapy regimens^8,9^, and monitoring for molecular residual disease (MRD) during follow-up has the potential to identify relapse earlier than would be detected with routine clinical surveillance^1,2,5,10^.

ctDNA detection can be tumor-informed or tumor-agnostic. Tumor-informed approaches leverage information from genomic profiling of a tumor tissue specimen, allowing for tracking of tumor-specific mutations within plasma and typically improving sensitivity relative to tumor-agnostic approaches. In 2020, cancer personalized profiling by deep sequencing (CAPP-seq), was used in a tumor-informed approach to demonstrate that preoperative ctDNA detection in non-small-cell lung cancer (NSCLC) could be used to identify patients with stage I disease with poor clinical outcome^11^. Subsequent work within lung adenocarcinomas (LUADs) from the LUNGCA-1 cohort^12^, the NADIM trial^13^ and the TRACERx study^1^ of NSCLC confirmed the prognostic capacity of preoperative ctDNA detection for overall survival (OS) and relapse-free survival (RFS) in LUADs.

Detection of preoperative ctDNA in early-stage LUAD is a considerable challenge owing to the low levels of ctDNA in plasma, which are frequently below 100 ppm^10,14^. Additionally, the sensitivity of ctDNA detection can be impaired by variations in the production of cell-free DNA (cfDNA) by non-malignant cells^15^, sequencing error and variants arising from clonal hematopoiesis of indeterminate potential (CHIP), which can be present at low levels in plasma^16^. A high-quality ctDNA detection platform must have a number of attributes for optimal clinical utility: it must be extremely sensitive, highly specific and applicable to a broad spectrum of tumors, and it must deliver results with small amounts of DNA input. To that end, there has recently been significant focus on research into developing approaches to overcome this problem^6,17–20^.

Although the relationship between ctDNA detection and survival is independent of pathological tumor-node-metastasis (pTNM) stage in this setting, the degree to which the limit of detection (LOD) of ctDNA assays affects the clinical sensitivity of ctDNA as a biomarker for aggressive disease is not well understood.

**Fig. 1:**
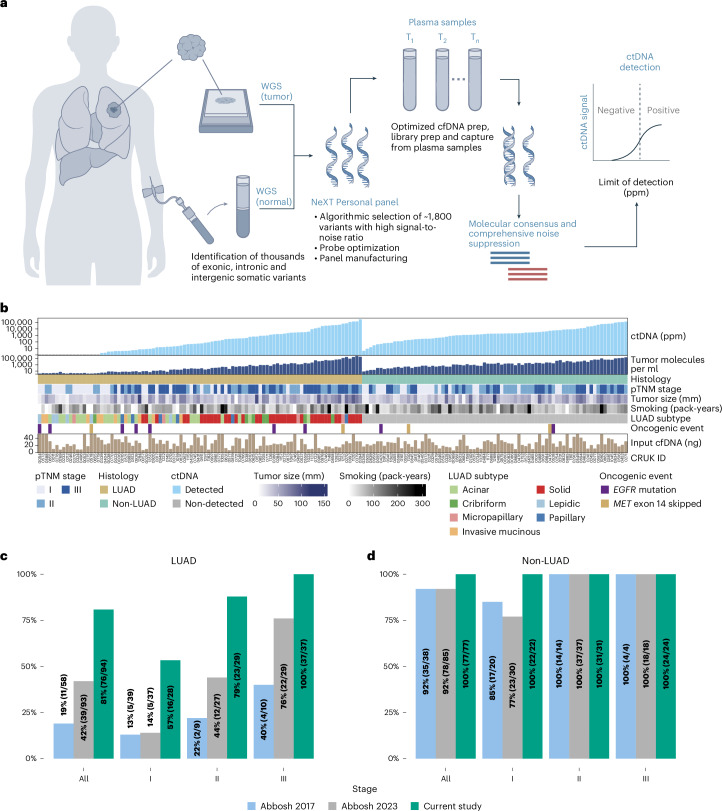
Highly sensitive detection of preoperative ctDNA. **a**, The NeXT Personal platform leverages tumor-informed information to achieve ultrasensitive and specific residual and recurrent cancer detection, longitudinal monitoring and therapy monitoring from liquid-biopsy samples. **b**, Clinicopathological variables relating to preoperative ctDNA detection in patients with NSCLC in the TRACERx study: ctDNA level (ppm tumor fraction); number of tumor molecules per ml plasma; pathological tumor node metastasis (pTNM) stage; NSCLC histology; tumor size (pathology-based tumor size (mm)); cigarette smoking (pack-years); pathological subtype of LUAD; presence of an oncogenic event (within this cohort, either the presence of an EGFR mutation or skipping of MET exon 14); and cfDNA input amount (ng). *n* = 171. **c**,**d**, Fraction of TRACERx LUAD (**c**) and non-LUAD (**d**) tumors detected pre-operatively. Colors represent different studies: blue, Abbosh et al.^1^; gray, Abbosh et al.^2^; green, this study. *n* = 94 LUAD, *n* = 77 non-LUAD.

Here, we used NeXT Personal, an ultrasensitive, tumor-informed liquid-biopsy platform to characterize preoperative ctDNA in 171 patients in the TRACERx study^1,21,22^. NeXT Personal is a tumor-informed liquid-biopsy platform that leverages prioritized target selection from whole-genome sequencing of tumor and matched normal DNA^23^. The development and analytical validation of this assay is outlined in Methods and in ref. ^23^. In brief, the method aims to achieve a LOD approaching 1 ppm by aggregating the signal from a larger number of somatic variant targets than can be detected from an exome. To avoid being overwhelmed by false signals arising from the large number of variants, noise must be suppressed to very low levels, which is largely accomplished by molecular consensus, which allows identification of independent sequence reads arising from a common founder, and groups these reads into unique molecule families for further analysis. NeXT Personal bespoke panels are designed using the top ~1,800 signal-to-noise ranked somatic variants for ctDNA detection from plasma (that is, the subset of cfDNA that contains the tumor-specific mutations in the panel). Hybridization-based genomic-target enrichment using the panel is followed by ultradeep sequencing of the plasma samples. NeXT Personal then aggregates the tumor-derived signal from the somatic targets. This process, combined with comprehensive noise-suppression methods, enables NeXT Personal to achieve ultrasensitive ctDNA detection for disease stratification, therapy monitoring and MRD detection (Methods and Fig. 1a).

Personalized tumor-informed ctDNA-detection assays that leverage exonic mutations have been investigated in the TRACERx cohort^1,2^. We have previously studied the ability of ctDNA, detected in a preoperative peripheral blood sample, to predict clinical outcome in LUAD^1^. This involved a tumor-informed assay investigating somatic variants at an average of 200 positions per sample, revealing that patients with LUAD who had ctDNA detected in their blood at the time of surgery had a worse clinical prognosis^24^. However, ctDNA was detectable in only 14% of patients with pathological stage I LUAD at this time point. We therefore set out to assess the degree to which a more sensitive and specific assay would increase prognostic value in a cohort with comparable clinical demographics (Extended Data Table 1).

**Extended Data Fig. 1:**
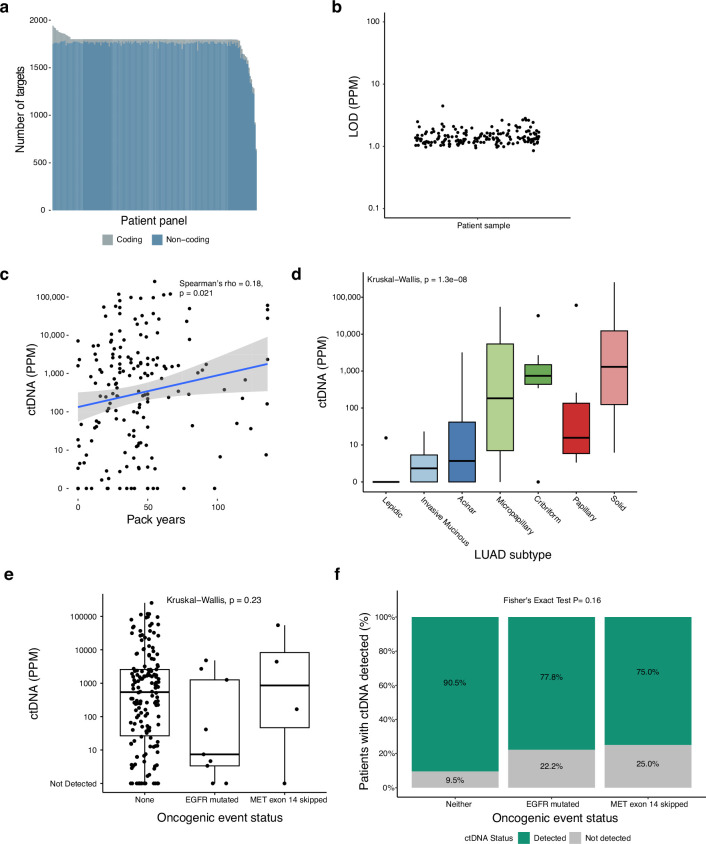
Association between ctDNA and clinicogenomic features of NSCLC. **a**. Stacked barplot of the number of targets obtained from coding (grey) and noncoding (blue) regions of the genome for each panel. **b**. Dot plot depicting panel-specific limit of detection (LOD) in ppm in all pre-operative plasma samples. **c**. Scatterplot demonstrating the association of ctDNA level with pack years of smoking for preoperative samples. Fitted line represents a linear model, and the shaded area represents the 95% confidence interval. **d**. Boxplot of preoperative ctDNA level for each pathological subtype of lung adenocarcinoma. The boxplots depict the median at the middle line, the lower and upper hinges represent the first and third quartiles, respectively, the whiskers show minima to maxima no greater than 1.5× the interquartile range (IQR), with the remaining outlying data points plotted individually. Sample size is n = 94 patients. *P* value was calculated using two-sided Kruskal-Wallis rank sum test. **e**. Boxplot of preoperative ctDNA level by oncogenic event status. The boxplots depict the median at the middle line, the lower and upper hinges represent the first and third quartiles, respectively, the whiskers show minima to maxima no greater than 1.5× the IQR, with the remaining outlying data points plotted individually. Sample size is n = 171 patients. *P* value was calculated using two-sided Kruskal-Wallis rank sum test. **f**. Barplot of patient oncogenic event status colored by preoperative ctDNA detection status. *P* value was calculated using two-sided Fisher’s exact test.

We analyzed blood plasma samples collected before the surgical removal of lung cancer from 171 TRACERx patients, including 94 with LUAD (29.8% stage I, 30.9% stage II, 39.3% stage III) and 77 with non-LUAD (28.6% stage I, 40.3% stage II, 31.2% stage III) NSCLC, using NeXT Personal (Extended Data Table 2 and Fig. 1b). Of these patients, 160 had one primary NSCLC tumor and 11 had two synchronous primary NSCLC tumors. A median of 1,800 patient-specific somatic variants were included in the NeXT Personal panel design (range, 646–1,942), of which a median of 97.83% were from non-coding regions (Extended Data Fig. 1a). This resulted in a set of bespoke panels with a median predicted LOD of 1.33 ppm and a range of 0.85–4.45 ppm (Extended Data Fig. 1b). The median DNA input quantity was 23.5 ng (Fig 1b; range, 4.01–50.0 ng).

ctDNA was detected in a preoperative plasma sample in 81% of patients with LUAD (Fig. 1c, 76/94) and 100% of patients with non-LUAD (Fig. 1d, 77/77) NSCLCs across a broad range of tumor fractions (positive ctDNA detection range, 1.66–253,826 ppm). This included 32 LUADs (34% of all LUADs) in which ctDNA was detected, but at below 80 ppm, the 95% LOD in our previous approach^2^. We could detect ctDNA in the blood of 57% of patients with pTNM stage I LUADs (16/28): ctDNA from these tumors has been difficult to detect in blood samples (only 14% of such tumors were identified in Abbosh et al.^2^ and 13% in Abbosh et al.^1^). Similarly, ctDNA was detected in 79% of pTNM stage II LUADs (23/29, compared to 44% in Abbosh et al.^2^). ctDNA shedding, as previously reported, was associated with smoking status (pack-year history) (Spearman’s *⍴* = 0.18, *P* = 0.021; Extended Data Fig. 1c)^2^ and with the high-grade predominant subtypes of LUAD, in particular the solid and cribriform subtypes (*P* = 1.3 × 10^–8^, Kruskal–Wallis test; Extended Data Fig. 1d)^25^. Oncogenic events, which in this cohort comprised *EGFR* mutations and skipping of *MET* exon 14 (no RET-ROS1-ALK oncogenic fusions were detected), were not associated with a significant difference in ctDNA ppm level (*P* = 0.23, Kruskal–Wallis test; Extended Data Fig. 1e) or rate of preoperative ctDNA detection (*P* = 0.16, Fisher’s exact test; Extended Data Fig. 1f), although this analysis is likely to have been underpowered given the small numbers of patients harboring these events.

**Fig. 2:**
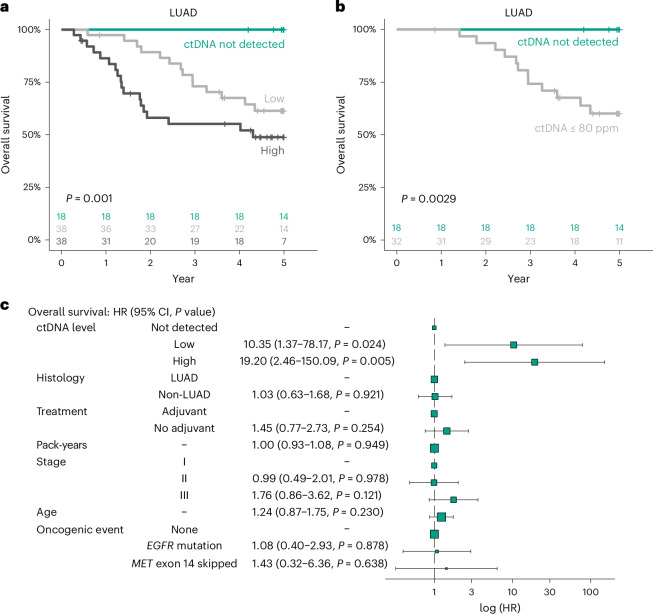
Baseline ctDNA level is prognostic of OS. **a**, Kaplan–Meier (KM) curve of OS in ctDNA-high (dark gray), ctDNA-low (light gray) and ctDNA-negative (green) patients with LUAD. ctDNA-high and ctDNA-low groups were defined according to the median ctDNA levels across ctDNA-positive LUADs. *P* values were calculated using log-rank tests. **b**, KM curve demonstrating OS in patients harboring ctDNA at an estimated tumor fraction below the limit of reliable detection described in Abbosh et al.^2^ (light gray) and ctDNA-negative patients (green). *P* values were calculated using log-rank tests. **c**, Results of multivariable Cox regression analysis including ctDNA level (ctDNA-high, ctDNA-low, ctDNA-negative); histology; whether the patient received adjuvant chemotherapy; cigarette smoking history (in increments of 10 pack-years); pTNM stage; age (in increments of 10 years); and the presence of an oncogenic event (either an *EGFR* mutation or *MET* exon 14 skipping). *n* = 171. Error bars represent 95% confidence intervals. The size of the boxes represent the number of patients within each category.

**Extended Data Fig. 2:**
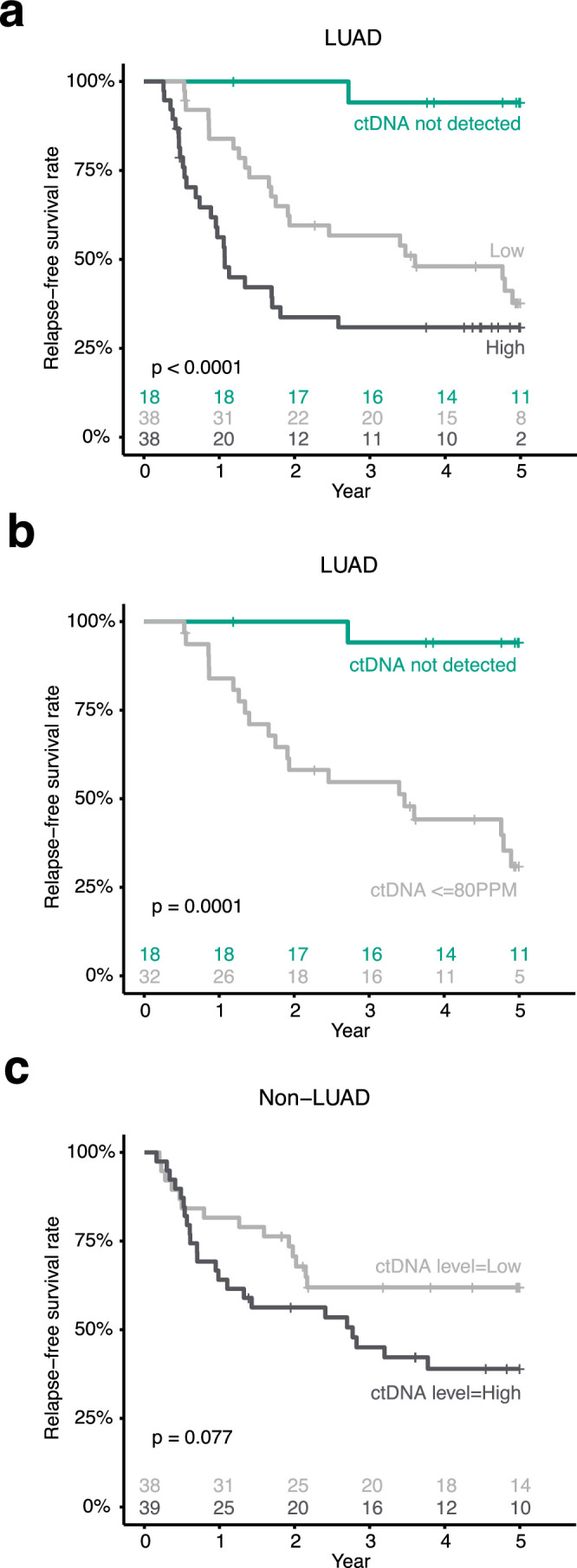
Increased assay sensitivity improves stratification of relapse-free survival. **a**. Kaplan–Meier (KM) curve demonstrating relapse-free survival (RFS) within ctDNA-high (dark grey), ctDNA-low (light grey) and ctDNA-negative (green) patients with lung adenocarcinoma. ctDNA high and low groups were defined according to the median ctDNA levels across ctDNA-positive LUADs. P values were calculated using log-rank tests. **b**. KM curve demonstrating RFS within patients harbouring ctDNA at an estimated ppm below the limit of reliable detection described in Abbosh et al. 2023^2^ (light grey), and ctDNA negative patients (green). P values were calculated using log-rank tests. **c**. KM curve illustrating difference in RFS between ctDNA-high and ctDNA-low patients with non-LUAD. P values were calculated using log-rank tests.

We next assessed the degree to which this additional sensitivity improved our ability to stratify these patients according to clinical outcome. Preoperative ctDNA-negative patients were compared with patients whose ctDNA levels were below the median of those detected (ctDNA-low) and those with ctDNA levels above the median of those detected (ctDNA high). ctDNA status predicted OS in LUADs (Fig. 2a, low: hazard ratio (HR) = 11.08, 95% confidence interval (CI) = 1.48–83.2; high: HR = 19.33, 95% CI = 2.56–146.0) and RFS (Extended Data Fig. 2a, low: HR, = 14.17, 95% CI = 1.91–105.3; high, HR = 25.79, 95% CI = 3.48–191.4). Patients with a preoperative ctDNA-negative status had significantly improved OS (5-year OS, 100%; 95% CI = 100%–100%; *n* = 18) compared with ctDNA-low patients (5-year OS, 61.4%; 95% CI = 47.3%–79.6%; *n* = 38), and ctDNA-high patients (5-year OS, 48.8%; 95% CI = 34.7%–68.7%; *n* = 38). Notably, when analysis was restricted to include only patients in whom ctDNA would not have been reliably detected using the approach in Abbosh et al.^2^, the presence of ctDNA at levels below 80 ppm remained prognostic for poor OS (Fig. 2b, *P* = 0.0029; HR = 12.33; 95% CI = 1.63–93.35) and RFS (Extended Data Fig. 2b, *P* = 0.00011; HR = 18.07; 95% CI = 2.41–135.3) in LUAD. This suggests that clinically meaningful signal is detected by assays with sensitivity at tumor fractions below 80 ppm, and that the ultra-high-sensitivity assay presented here enables identification of a group of very-low-risk patients with LUAD.

As we have previously reported, the association between outcomes and elevated preoperative ctDNA levels in non-LUADs was substantially reduced compared with that in LUADs; previous work has found no discernible impact of ctDNA levels on clinical outcome in non-LUADs^1^. In this analysis, a ctDNA level greater than the median in non-LUADs was not associated with reduced RFS (Extended Data Fig. 2c; HR = 1.81; 95% CI = 0.93–3.92; *P* = 0.077). This highlights a fundamentally different relationship between ctDNA and disease biology in non-LUADs compared with that in LUADs.

**Extended Data Fig. 3:**
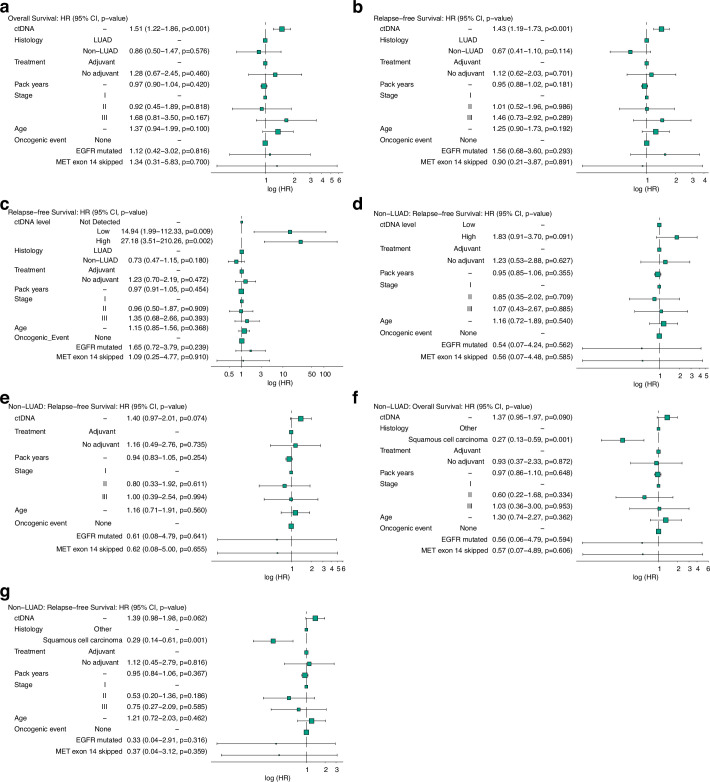
Multivariable analyses adjusting for known risk factors confirms independent prognostic value of pre-operative ctDNA. **a**. Multivariable Cox regression analysis for overall survival (OS) containing ctDNA (continuous, per 10-fold increase); histology; whether the patient received adjuvant chemotherapy; cigarette smoking status (in 10 pack year increments); pTNM stage; age (in 10 year increments); and the presence of an oncogenic event. n = 171 patients. **b**. Multivariable Cox regression analysis for relapse-free survival (RFS) containing ctDNA (continuous, per 10-fold increase); histology; whether the patient received adjuvant chemotherapy; cigarette smoking history; pTNM stage; age; and the presence of an oncogenic event. n = 171 patients. **c**. Multivariable Cox regression analysis for RFS containing ctDNA level (ctDNA-high, ctDNA-low, ctDNA-negative); histology; whether the patient received adjuvant chemotherapy; cigarette smoking history; pTNM stage; age.; and the presence of an oncogenic event. n = 171 patients. **d**. Multivariable Cox regression analysis for RFS in non-LUADs containing ctDNA level (ctDNA-high, ctDNA-low); whether the patient received adjuvant chemotherapy; cigarette smoking history; pTNM stage; age; and oncogenic events. n = 77 patients. **e**. Multivariable Cox regression analysis for RFS in non-LUADs containing ctDNA (continuous); whether the patient received adjuvant chemotherapy; cigarette smoking history; pTNM stage; age; and oncogenic events. n = 77 patients. **f**. Multivariable Cox regression analysis for OS in non-LUADs containing ctDNA (continuous, per 10-fold increase); sub-histology; whether the patient received adjuvant chemotherapy; cigarette smoking history; pTNM stage; age and oncogenic events. n = 77 patients. **g**. Multivariable analysis for RFS in non-LUADs containing ctDNA (continuous, per 10-fold increase); sub-histology; whether the patient received adjuvant chemotherapy; cigarette smoking history; pTNM stage; age; and oncogenic events. n = 77 patients. For all plots in this figure, error bars represent 95% confidence intervals. The size of the boxes represents the number of patients within each category.

When adjusted for histology, pTNM stage, smoking status, age, the presence of an oncogenic event (such as an *EGFR* driver mutation or the skipping of exon 14 in *MET*) and the addition of adjuvant therapy, the presence of ctDNA—whether considered either as a continuous metric or stratified into groups as specified above—was independently associated with reduced OS (Fig. 2c and Extended Data Fig. 3a) and RFS (Extended Data Fig. 3b,c) in a pooled cohort of patients with LUADs and non-LUADs. Of note, the ctDNA level was not significant as an independent prognostic factor for RFS in the non-LUAD group (Extended Data Fig. 3d,e). The prognostic effect of ctDNA as a continuous variable on both RFS and OS was not significant when adjusting for histological (squamous versus non-squamous) subtype and other clinicopathological factors (Extended Data Fig. 3f,g).

This work has leveraged NeXT Personal, a tumor-informed assay that is capable of reliably detecting ctDNA in blood at 1–3 ppm (0.0001–0.0003% tumor fraction). Notably, this ultra-high sensitivity can be achieved with an estimated specificity of 99.9% and even from suboptimal DNA input volumes. This could be important in many clinical settings, and can be applied to suboptimal DNA input volumes.

Patients with early-stage NSCLC remain at high risk of relapse, despite aggressive curative-intent treatment. Thus, it is of critical importance to accurately stratify patients to both maximize the likelihood of disease cure following surgery and adjuvant therapy and minimize risk of overtreatment in those patients predicted to have good outcome. Detectable preoperative ctDNA has been associated with worsened recurrence-free survival and reduced OS^12,26,27^, and has been suggested as a potential marker for neoadjuvant treatment selection^8,9^. We have demonstrated that assays that cannot detect ctDNA at tumor fractions below 80 ppm fail to capture a clinically impactful signal arising from a significant subset of patients with LUAD^2^. In this study, these patients with detectable but extremely low levels of ctDNA experienced a worse clinical outcome than those in whom we did not detect evidence of ctDNA. This suggests that there is a subset of very-low-risk patients with LUADs who can be definitively identified only by using an ultrasensitive ctDNA assay, raising the potential for an ultrasensitive and specific assay to be used prognostically for escalation of therapy in stage I LUADs exhibiting ctDNA release.

Although this study presents results from preoperative plasma samples, the high sensitivity of the NeXT Personal assay suggests the potential for significant clinical benefit in the setting of minimal residual disease for tracking treatment response and detecting recurrence.

Of note, there are a number of technologies in the field aiming to achieve ultrasensitive tumor-informed ctDNA detection, such those developed by Foresight Diagnostics (PhasED-seq)^18,28^, C2i Genomics^19^ and Inivata (RaDaR)^6^.

This work has limitations. The data from TRACERx was analyzed retrospectively, although in a blinded fashion. Future data from prospective cohorts will be needed to evaluate the clinical utility of this assay. Although NeXT Personal is already in use as a clinical diagnostic test, it, like other tumor-informed ctDNA detection assays, is of higher complexity, can be more costly to produce and requires a longer turnaround period to develop the panel and obtain a clinically actionable result, compared with non-tumor-informed approaches.

If ctDNA is to be used for clinical risk prediction, the design of personalized adjuvant treatment regimens and early detection of recurrence and integrated into routine clinical care, ctDNA assays should have a high degree of sensitivity. In this way, they hold promise to transform adjuvant clinical trial design and clinical practice.

## Methods

### Baseline characterization of lung cancer samples

**Extended Data Fig. 4:**
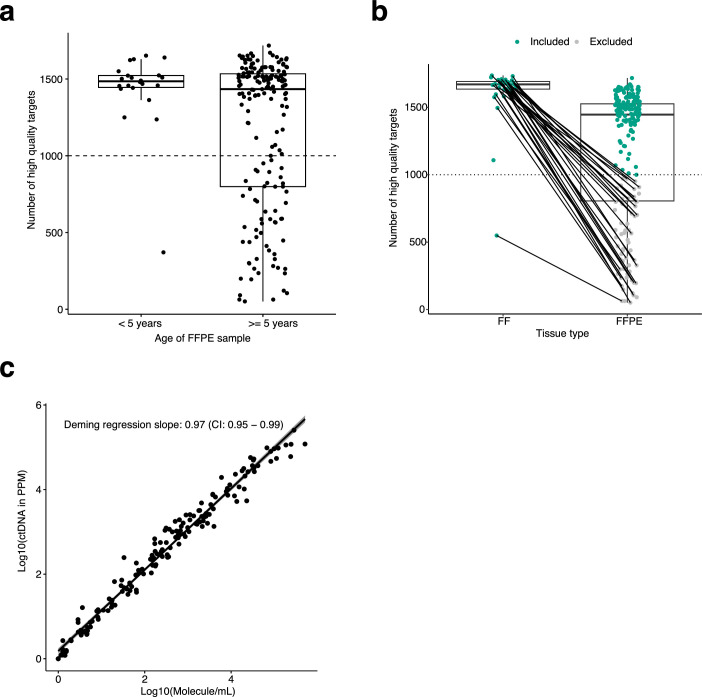
The impact of sample age and preservation format on panel targets. **a**. Box and dot plot comparing number of high quality targets per panel from formalin-fixed paraffin embedded (FFPE) samples greater than 5 years vs less than 5 years old. Horizontal dashed line at 1000 indicates QC threshold. Center line represents the median. The upper whisker is the maximum value of the data that is within 1.5 times the interquartile range over the 75th percentile. The lower whisker is the minimum value of the data that is within 1.5 times the interquartile range under the 25th percentile. Sample size is n = 202. **b**. Failed FFPE or FFPE-derived panels which yielded fewer than 1000 high quality targets were re-generated using fresh frozen (FF) tissue where available. Green dots indicate samples included in our analysis, grey dots indicate those that failed to attain sufficient high quality targets for further processing. Where possible these panels were replaced with FF-derived panels. Connecting lines indicate paired patient panels designed from either FF or FFPE tumor sample. Center line represents the median. The upper whisker is the maximum value of the data that is within 1.5 times the interquartile range over the 75th percentile. The lower whisker is the minimum value of the data that is within 1.5 times the interquartile range under the 25th percentile. Sample size is n = 233 panels. One panel constructed from an FF tumor sample failed to attain 1,000 high-quality targets but was included to ensure that the cohort was representative of the wider TRACERx cohort. Two FF panels were constructed where FFPE panels had failed altogether; these are represented by green dots on the FF side not connected to an FFPE dot. **c**. Deming regression demonstrating agreement between measured ctDNA PPM and tumor molecules per milliliter of plasma. Fitted line represents a linear model, and the shaded area represents the 95% confidence interval.

TRACERx patient recruitment and sample collection complied with all relevant ethical regulations and were carried out as previously described^2^. The eligibility criteria have been previously described^2^. Of note, the eighth version of pTNM staging was used in this analysis. Patients with histopathologically confirmed stage I–IIIB NSCLC who were eligible for primary surgery were enrolled in the prospective observational TRACERx study (ClinicalTrials.gov identifier: NCT01888601). The study design was approved by an independent research ethics committee (NRES Committee London, REC ref. 13/LO/1546), and informed consent was obtained from all patients before study admittance. Patient sample identifiers were anonymized and tracked in a centralized database controlled by the study sponsor. DNA degradation of archived formalin-fixed paraffin-embedded (FFPE) samples over time can affect panel quality for ctDNA detection, and degraded samples might not accurately reflect the typical sample quality in a clinical setting in which FFPE samples were recently collected (Extended Data Fig. 4a). We obtained FFPE tissue for 204 patients. Of these, 62 had atypically low counts of high-quality panel targets (<1,000), likely owing to age and/or poor quality of the FFPE samples, and 2 did not pass panel design. For 31 of the 64 patients, DNA extracted from fresh frozen (FF) tissue was available by September 2023. For these samples, an updated set of panels was generated, the quality of which was more consistent with <5-year-old FFPE samples (Extended Data Fig. 4b). One panel was constructed from an FF tumor sample and failed to attain 1,000 high-quality targets; however, this was included to ensure that the cohort was representative of the wider TRACERx cohort. Two FF panels were constructed where FFPE panel construction had failed entirely. The final cohort in our study consisted of preoperative samples from 171 consecutively recruited patients in the larger TRACERx study in whom adequate plasma was available for the ctDNA analysis completed by September 2023, in addition to confirmed clinical outcome data as of February 2024. Retrospective ctDNA analysis was conducted using prospectively collected specimens and during clinical follow-up. In this cohort, we demonstrated that our measure of ctDNA signal (ppm) was in strong agreement with tumor molecules per ml plasma, which accounts for the volume of plasma from which input cfDNA is extracted (Demming regression; fitted slope = 0.97, CI = 0.95–0.99; Extended Data Fig. 4c). Personalis investigators were fully blinded to patient clinical outcome and clinical pathological characteristics during sample processing and ctDNA analysis. Likewise, TRACERx investigators were blinded to patient ctDNA status during clinical data and patient specimen collection. *EGFR* mutations, oncogenic fusion isoforms and instances of *MET* exon 14 skipping from patients in the TRACERx cohort were annotated as previously described^29^.

### Tumor and normal whole-genome sequencing

Tumor sections were macrodissected to improve tumor content and were required to meet a tumor cellularity threshold of ≥20%, as determined by pathological review, to be eligible for DNA extraction and further processing. At this threshold, 1.2% (2/171) of samples were considered ineligible for analysis and required replacement with different specimens. At our chosen 20% cut-off threshold, we observed no significant correlation between tumor purity and the LOD of the assay (Supplementary Fig. 1). Genomic DNA was isolated from matched tumor and normal samples using the Qiagen AllPrep DNA/RNA FFPE Tissue Kit or the QIAamp DNA Mini Kit (Qiagen) using internally optimized workflows. Whole-genome sequencing (WGS) libraries were prepared with 100–500 ng of acoustically sheared genomic DNA (Covaris) using the KAPA HyperPrep Kit (Roche Sequencing Solutions) and customized methods. Libraries were cleaned up using AMPure XP beads and then quantified using the KAPA Library Quantification Kit (Roche Sequencing Solutions), before being sequenced to ×30 depth of coverage using a NovaSeq 6000 instrument (Illumina). The impact of varying DNA input amounts during tumor WGS on panel design was assessed using 19 normal–tumor pairs. For each pair, normal libraries were made using 550 ng input DNA, and tumor libraries with 5 ng, 15 ng, 50 ng, 200 ng or 550 ng of input DNA. We observed largely consistent panel size and similarity across the range of input DNA amounts (Supplementary Fig. 2a,b). A comprehensive list of reagents used in this study is provided in Supplementary Table 1.

### Alignment and variant calling from tumor and normal whole-genome sequencing

The pipeline performs alignment, duplicate removal and base quality-score recalibration (BQSR) of the matched tumor and normal WGS samples using best-practice guidelines recommended by the Broad Institute^30,31^. In brief, individual read-pairs were first mapped to the hs37d5 reference genome build using the BWA–MEM aligner. We then used the Picard toolkit (RRID: SCR_006525) to identify duplicate reads through comparison of the 5′ position of reads and read-pairs. Duplicate reads were then removed. The Genome Analysis Toolkit (GATK, RRID: SCR_001876) was then used for sequence realignment and to apply base quality scores (BQSR): the BaseRecalibrator tool uses the deduplicated data and a set of known variants to construct a model of covariation, which is the used to generate a recalibration file. The ApplyBQSR tool then uses this model to adjust base quality scores in the data, yielding a new BAM file. Aligned sequence data are written in BAM format according to SAM (RRID: SCR_01095) specification. MuTect (RRID: SCR_000559) was used to co-analyze the tumor and normal BAM files for somatic single-nucleotide variant (SNV) detection. Somatic SNV calls were filtered on the basis of a broad set of quality-control metrics, such as local sequence coverage and read quality, strand bias and the statistical likelihood that the allele is present in the normal sample.

### NeXT personal probe panel design

Hybrid capture probe panels used in this study were designed with the NeXT Personal platform’s proprietary algorithms, as governed by the standard operating procedures at Personalis. In the design process for the bespoke panel, for each patient’s panel, the ctDNA targets were selected from exonic, intronic and intergenic somatic variants identified through WGS of matched tumor and normal samples, as described above. Somatic variants identified using Mutect (v1.1.6, default parameters) were selected and assigned an error rate according to the observed substitution in the solid tumor. Namely, the substitution error rate was estimated by the ratio of the aggregated amount of signal and the total number of molecules observed in each possible substitution in more than 200 healthy plasma samples^23^. The MRD targets were then selected from somatic variants with an allele frequency above 10%. Variants were further filtered by excluding those found in particular regions of the genome. Exclusion criteria included regions containing known germline SNPs, known CHIP variants, high GC content (≥80%), high polymorphism rates, mapping difficulties, systematic bias, short tandem repeats and low sequence complexity^32^.

Somatic variant calls were ranked by the product of allele frequency in solid tumor and the substitution-based error rate of solid tumor substitutions. Up to ~1,800 top ranked somatic variants were selected genome-wide for panel inclusion by the NeXT Personal platform. The final panel also included 43 population SNVs for quality-assurance purposes (that is, to detect potential sample–panel mismatch or contamination). Several criteria were used to optimize the selection of the 43 SNVs, including having a population frequency of at least 20%, being in Hardy–Weinberg equilibrium and being outside of the HLA region. The SNPs were prioritized to have roughly equivalent representation across subpopulations. Probe sequences were designed by the NeXT Personal platform’s proprietary algorithm before being processed for manufacturing. Upon receiving panel reagents, the new panel was used for targeted sequencing of blood plasma from an unrelated healthy donor. This served two purposes: quality-control tests on the sequencing data were used to qualify the panel for use on the patient’s plasma samples, and any MRD targets for which any non-reference signal was observed were deactivated in the logical panel design, to mitigate the risk that those targets could be enriched for noise.

### NeXT Personal cfDNA library preparation, target enrichment and sequencing

Library preparation, target enrichment and sequencing of the cfDNA samples were performed in CLIA-certified and CAP-accredited laboratories, as governed by the standard operating procedures at Personalis. In brief, sequencing libraries were prepared from 2.45–50 ng cfDNA input (median, 15 ng), using the KAPA HyperPrep Kit (Roche Sequencing Solutions) and customized methods. We assessed the effect of the quantity of total input cfDNA on MRD detection and ctDNA burden and observed no significant associations between the amount of cfDNA input and ctDNA detection status (Supplementary Fig. 3). Consistently, there was no significant correlation between the quantity of input cfDNA and ctDNA burden or ctDNA burden and the LOD of the assay (Supplementary Fig. 3b,c). Together, this evidence suggests that ctDNA burden and detection status were not confounded by the quantity of total circulating DNA in the cohort. These findings are supported by a separate analytical validation study^23^. The pre-capture libraries were quantified using a Lunatic spectrophotometer (Unchained Labs), and up to 1,500 ng was enriched with patient-specific NeXT Personal probe panels using proprietary modifications to the Fast Hybridization and Wash Kit (Twist Bioscience) and workflow. The postcapture libraries were then amplified by PCR (nine cycles), and a quality assessment was performed using the TapeStation system (Agilent Technologies). The final libraries were cleaned up using AMPure XP beads and then quantified using the KAPA Library Quantification Kit (Roche Sequencing Solutions) before being sequenced on a NovaSeq 6000 instrument (Illumina). The libraries were deeply sequenced to optimize the number of unique observed molecules. We observed a weak correlation between sequencing depth and the LOD for each custom assay, as well as between sequencing depth and the strength of ctDNA signal detected (ppm level); however, the ctDNA detection status (detected or not detected) was not confounded by the variability of sequencing depth, with no significant difference in sequencing depth observed between the two groups (Supplementary Fig. 4a–c).

### NeXT Personal cfDNA analysis

Analysis of all NeXT Personal data in this study was performed using a consistent, locked version of the production pipeline developed by Personalis. In summary, the cfDNA sequencing data were aligned to the human reference genome (version hs37d5), followed by noise suppression and ctDNA detection. More specifically, we built and filtered the molecular consensus as follows. First, we aligned all reads to the human reference using BWA–MEM (Burrows–Wheeler Aligner, v1.0.2). Second, we grouped read-pairs according to their paired mapped positions to form initial consensus groups. With a positional approach like this, there is a risk of grouping multiple molecules together that share paired mapped positions. We mitigated this risk by detecting the presence of non-reference alleles that were present in not only a subset of the consensus-group reads, but also at least two other consensus groups. When there was an allele present in a subset of reads with additional support from other consensus groups, we split the consensus group to isolate the allele-containing reads in their own new group. For each group, we required observation of at least one molecule from each DNA strand. Raw reads that differed by more than 2.5% across the consensus molecule were not included. Bases with a quality of less than 29 were masked. Once we defined the consensus groups, we formed a single consensus molecule from the reads in each group on the basis of identification of the consensus basecall at each position along the group of read-pairs. Bases with less than 90% agreement in the molecular group were masked. Reads with more than 20% of their bases masked were removed. Then, we re-mapped these consensus reads again using BWA–MEM to avoid any erroneous alignment caused by sequencing errors. Following noise suppression, tumor-derived signal was aggregated in a tested sample across ctDNA targets in each patient-specific panel to calculate the ctDNA level (measured in ppm, based on the total unique molecule count). A one-tailed Poisson test was then performed to determine ctDNA detection status for each tested sample. The observed aggregate tumor-derived signal across each panel serves as the tested value, with the expected noise arising from accumulated background error being set as the mean of the Poisson distribution. The *P* value threshold was established as previously described^23^. In brief, the *P* value threshold is set at <0.001 to ensure that an analytical specificity requirement of >99.9% was met. Therefore, the *P* value threshold was set to 0.001 for this study to ensure higher specificity. If the tumor signal was significantly (*P* ≤ 0.001) above the expected noise, the sample was classified as ctDNA-positive (that is ‘detected’); otherwise, it was classified as ctDNA-negative (that is ‘not detected’). Given that the *P* value is the probability that the observed signal comes from noise, the detection threshold is set to enforce a specificity requirement and is independent of factors that affect observed levels. Variations in assay and locus-specific factors might affect the efficiency of detection of a specific genetic alteration. Different genetic alterations could also be present at different frequencies in the blood. The actual ppm level (allele frequency) measured is a function of the set of targets selected. After aggregation across many loci, however, the average per-locus efficiency of detection tends toward the population mean of the efficiency of detecting a specific genetic alteration. This is demonstrated in Supplementary Figure 5a, which shows that, as the panel size approaches 1,800 targets, the coefficient of variation (CV) of the observed ppm level adds little to the overall variability of the assay. Basing detection status on a *P* value, rather than an allele frequency threshold, allowed our approach to normalize detection efficiency for specific mutations through the summation of signal across up to 1,800 variant loci that have been selected on the basis of their site-specific error rates, inherent noise and complexity.

### Clonal hematopoiesis of indeterminate potential

We designed our assay to prevent CHIP mutations from being included in the bespoke panel by taking a tumor-matched-normal approach for somatic variant calling to inform panel design. This is an effective approach because the CHIP signal is higher in the normal blood cells than in tumor tissue, and thus will be filtered out in the tumor normal algorithmic comparison. We also excluded the most common CHIP regions from our panel design.

### TRACERx cfDNA extraction and quantification

Blood samples were collected in K_2_-EDTA tubes. Samples were processed within 2 h of collection by double centrifugation of the blood, first for 10 min at 1,000*g*, then the plasma for 10 min at 2,000*g*. Plasma was stored in 1-ml aliquots at −80 °C. Following isolation, plasma was shipped on dry ice. At the time of analysis, TRACERx plasma samples were between 2 and 9 years old. Up to 24 h before cfDNA extraction, the plasma was thawed and aliquots from the same patient plasma time point were consolidated and then stored at 4 °C. Immediately before cfDNA extraction using QIAamp Circulating Nucleic Acid or QIAsymphony Circulating DNA kits (Qiagen), consolidated plasma was clarified at 16,000*g* to remove cryoprecipitates.

### Samples used for DNA input performance characterization

All experiments were performed in the Clinical Laboratory Improvement Amendments (CLIA)-certified and College of American Pathologists (CAP)-accredited laboratories at Personalis, as guided by the Association for Molecular Pathology (AMP) and CAP’s joint recommendations^33^. Healthy donor and patient tissue and matched buffy-coat and plasma samples used in Supplementary Figures 2 and 5 were sourced from either Boca Biolistics, Cureline or iProcess. Patient samples in this study obtained from commercial vendors were collected from informed patients following receipt of their written consent under study protocols approved by an Independent Ethical Committee or Institutional Review Board (Protocol numbers: PG-ONC 2003/1; IRB7 - Registration 5136; IRB 800959).

### Survival analyses

OS was defined as the days from registration to death or loss of follow-up. RFS was defined as the days from registration to any disease recurrence, new primary tumor events or death. Exploratory analysis comparing OS and RFS at different ctDNA levels was performed for a total 171 patients shown in Figure 2 and Extended Data Figure 3. Survival (3.3–1), survminer (0.4.9) and finalfit (1.0.4) R packages were used to generate hazard ratios, CIs, 2-year survival probability, forest plots, KM plots and Cox regression models. Differences in OS or RFS between different groups of patients were assessed using log-rank tests. The association of OS or RFS with continuous variables, such as ctDNA level, was assessed through Cox regression modeling. The independent prognostic value of ctDNA in either the continuous or categorical form was assessed by multivariable Cox regression models that included histology, adjuvant treatment status, smoking status, pathological stage and age.

### Statistical analysis and data handling

No statistical methods were used to predetermine sample size. Analysis was performed in the R statistical environment (4.1.3). All statistical tests were two-sided, unless stated otherwise. For assay-performance analyses, positive predictive value was calculated as all true-positive results divided by the sum of true-positive and false-positive results; negative predictive value was calculated as all true-negative results divided by the sum of false-negative plus true-negative results; sensitivity was calculated as true-positive results divided by the sum of true-positive and false-negative results; and specificity was calculated as true negatives divided by the sum of true negatives and false positives. For input and output operations and general data manipulation, the R packages tidyverse (v1.3.2) and lubridate were used (v1.9.2). For general visualization, the R packages ggplot2 (v.3.4.2), ggpubr (v.0.4.0), scales (v.1.2.1.) and ggnewscale (v.0.4.9) were used. For statistical analyses and related visualization, R packages survival (v.3.3–1), survminer (v.0.4.9), finalfit (v.1.0.4), gt (v.0.10.1) and mcr (v.1.2.2) were used.

### Reporting summary

Further information on research design is available in the Nature Portfolio Reporting Summary linked to this article.

## Online content

Any methods, additional references, Nature Portfolio reporting summaries, source data, extended data, supplementary information, acknowledgements, peer review information; details of author contributions and competing interests; and statements of data and code availability are available at 10.1038/s41591-024-03216-y.

## Supplementary information

Supplementary Information Supplementary Figures 1–5 and Supplementary Table 1

Reporting Summary

## Data Availability

Processed TRACERx patient data have been deposited on Zenodo at 10.5281/zenodo.8400837 (ref. ^34^). Supporting data from validation experiments are included as Extended Data Table 1. Raw data from TRACERx patients analyzed in this study, including fastq and bam files from tumor and normal WGS, as well as fastq files from cfDNA, have been deposited at the European Genome–phenome Archive (EGA), hosted by The European Bioinformatics Institute (EBI) and the Centre for Genomic Regulation (CRG) under the accession codes EGAS00001006494, under controlled access.

## Extended data

**Extended Data Table 1:** Baseline demographic and tumor characteristics for the study population split by lung adenocarcinoma and non-adenocarcinoma populations. Data are *n* (%), unless otherwise stated. Percentages may not sum to 100% due to rounding

Characteristic	levels	LUAD	Non-LUAD	p
n	-	94	77	NA
Age (years)	Mean (SD)	67.2 (8.9)	70.1 (8.0)	0.028
Sex	Female	40 (42.6)	29 (37.7)	0.623
	Male	54 (57.4)	48 (62.3)	
Pathological TNM	1a	11 (11.7)	6 (7.8)	0.718
	1b	17 (18.1)	16 (20.8)	
	2a	4 (4.3)	4 (5.2)	
	2b	25 (26.6)	27 (35.1)	
	3a	35 (37.2)	22 (28.6)	
	3b	2 (2.1)	2 (2.6)	
Smoking status	Ex-Smoker	41 (43.6)	46 (59.7)	0.086
	Never Smoked	6 (6.4)	2 (2.6)	
	Smoker	47 (50.0)	29 (37.7)	
Adjuvant treatment	Adjuvant	46 (48.9)	36 (46.8)	0.896
	No adjuvant	48 (51.1)	41 (53.2)	
LUAD subtype	Lepidic	6 (6.4)		
	Papillary	6 (6.4)		
	Acinar	23 (24.5)		
	Cribriform	7 (7.4)		
	Micropapillary	4 (4.3)		
	Solid	38 (40.4)		
	Invasive mucinous	10 (10.6)		
Oncogenic event	None	85 (90.4)	73 (94.8)	0.364
	EGFR mutated	7 (7.4)	2 (2.6)	
	MET exon 14 skipped	2 (2.1)	2 (2.6)	
Follow up (days; OS)	Median (# of events)	1839 (39)	1862 (38)	0.84
Follow up (days; RFS)	Median (# of events)	1821 (49)	1849 (40)	0.58

*P* values were calculated using chi-squared tests for categorical variables and *t* tests for continuous variables.

**Extended Data Table 2:** Baseline demographic and tumor characteristics for the current study presented alongside general characteristics of patients included in the work detailed in refs. ^1,2^. Data are *n* (%), unless otherwise stated. Percentages may not sum to 100% due to rounding

Characteristic	levels	Abbosh et al. 2023	Abbosh et al. 2017	NeXT Personal
Age (years)	Mean (SD)	68.8 (9.4)	68.4 (9.3)	68.5 (8.6)
Sex	Female	79 (40.9)	36 (37.5)	69 (40.4)
	Male	114 (59.1)	60 (62.5)	102 (59.6)
Adjuvant treatment	Adjuvant	77 (39.9)	28 (29.2)	82 (48.0)
	No adjuvant	116 (60.1)	68 (70.8)	89 (52.0)
Histology	Adenocarcinoma	101 (52.3)	57 (59.4)	94 (55.0)
	Other	25 (13.0)	8 (8.3)	20 (11.7)
	Squamous cell carcinoma	67 (34.7)	31 (32.3)	57 (33.3)
LUAD subtype	Lepidic	4 (4.2)	10 (17.9)	6 (6.4)
	Papillary	11 (11.5)	4 (7.1)	6 (6.4)
	Acinar	33 (34.4)	21 (37.5)	23 (24.5)
	Cribriform	9 (9.4)	1 (1.8)	7 (7.4)
	Micropapillary	4 (4.2)		4 (4.3)
	Solid	24 (25.0)	14 (25.0)	38 (40.4)
	Invasive Mucinous	11 (11.5)	6 (10.7)	10 (10.6)
Pathological TNM	IA	37 (19.2)	26 (27.1)	17 (9.9)
	IB	37 (19.2)	32 (33.3)	33 (19.3)
	IIA	40 (20.7)	14 (14.6)	8 (4.7)
	IIB	30 (15.5)	10 (10.4)	52 (30.4)
	IIIA	49 (25.4)	13 (13.5)	57 (33.3)
	IIIB		1 (1.0)	4 (2.3)
Smoking status	Ex-Smoker	103 (53.4)	49 (51.0)	87 (50.9)
	Never Smoked	10 (5.2)	11 (11.5)	8 (4.7)
	Smoker	80 (41.5)	36 (37.5)	76 (44.4)

## Code availability

Supporting code required to reproduce all analyses and figures included in this paper are available on Zenodo at 10.5281/zenodo.8400837 (ref. ^34^).
