# Supplementary material for: Ultrasensitive ctDNA detection for preoperative disease stratification in early-stage lung adenocarcinoma
Source: Nat Med. 2025 Jan 13;31(1):70–6. doi: 10.1038/s41591-024-03216-y (PMC11750713; doi:10.1038/s41591-024-03216-y)
Supplement: Supplementary file 1 — Supplementary Figures 1–5 and Supplementary Table 1 [file 41591_2024_3216_MOESM1_ESM.pdf]

nature medicine

Supplementary information https://doi.org/10.1038/s41591-024-03216-y

Ultrasensitive ctDNA detection for preoperative disease stratification in early-stage lung adenocarcinoma

In the format provided by the authors and unedited

Supplementary Figure 1: Pathologist estimates of tumour cellularity. a. Dot plot of patient-specific limit of detection (LOD) by pathologist estimated tumour cellularity in each patient's tumour sample. Pearson correlation coefficient and P value was calculated. n = 171.

Supplementary Figure 2: Patient ctDNA panel size is stable across a broad range of DNA input levels. a. Grouped dot plot depicting panel-specific MRD target counts derived from varying levels of tumour DNA input into WGS. Each color represents a single patient assayed over different levels of input. Sample size is n=82 from 19 patients. **b.** Box and dot plot comparing Jaccard similarity of targets per panel from 550ng input reference and diluted input amount into WGS. The height of boxes and whiskers represent the interquartile ranges (IQR: first and third quartiles) and 1.5 times the IQR of data, respectively. Each dot represents a single patient assayed at a certain level of input. Each color represents a single patient assayed over different levels of input. Sample size is n=62 samples from 19 patients.

Supplementary Figure 3: ctDNA detection status and level of signal are independent of cfDNA input amount. a. Box and dot plot comparing ctDNA detection status and cfDNA input amount. The box plots depict the median at the middle line, the lower and upper hinges represent the first and third quartiles, respectively, the whiskers show minima to maxima no greater than 1.5× the IQR, with the remaining outlying data points plotted individually. Sample size is n=171 patients. *P* value was calculated using the two-sided Wilcoxon rank sum test. **b.** Scatterplot demonstrating the association of ctDNA PPM level with cfDNA input amount. Fitted line represents a linear model, and the error band represents the 95% confidence interval. Pearson correlation coefficient and *P* value was calculated. Sample size is n=171 patients. **c.** Scatterplot demonstrating the association of ctDNA PPM level with patient-specific limit of detection. Fitted line represents a linear model, and the error band represents the 95% confidence interval. Spearman correlation coefficient and *P* value was calculated. Sample size is n=171 patients.

Supplementary Figure 4: Robust assay performance across a range of sequencing depths. a. Scatterplot demonstrating the association of assay limit of detection (LOD) with cfDNA sequencing depth. Fitted line represents a linear model, and the error band represents the 95% confidence interval. Color indicates MRD detection status. Spearman correlation coefficient and *P* value was calculated. Sample size is n=171 patients.**b.** Box and dot plot comparing sequencing depth between patients with MRD status (ctDNA detected vs. not detected). The height of boxes and whiskers represent the interquartile ranges (IQR: first and third quartiles) and 1.5 times the IQR of data, respectively. Dots represent patients with sequencing depth beyond 1.5 IQR. Sample size is n=171 patients. *P* value was calculated using a two-sided Wilcoxon

rank sum test. **c.** Scatterplot demonstrating the association of ctDNA PPM level with cfDNA sequencing depth. Fitted line represents a linear model, and the error band represents the 95% confidence interval. Pearson correlation coefficient and *P* value was calculated. Sample size is n=171 patients.

Supplementary Figure 5: The coefficient of variation of observed ctDNA level contributes little to assay variability. a. A single 1800 target panel was reanalyzed with the MRD targets broken up into equal-sized subsets totaling 1800. The number of subsets at each level is 1800 divided by the panel size, and each panel is completely orthogonal. The mean PPM (purple line) is the average of the measured PPMs for all panels of the same size. The coefficient of variation (CV; blue line) of the measured PPM is shown as a percent. The green line indicates the percentage of panels which yielded no signal at a given panel size.

Supplementary Table 1: List of reagents used during sample preparation and processing.

a

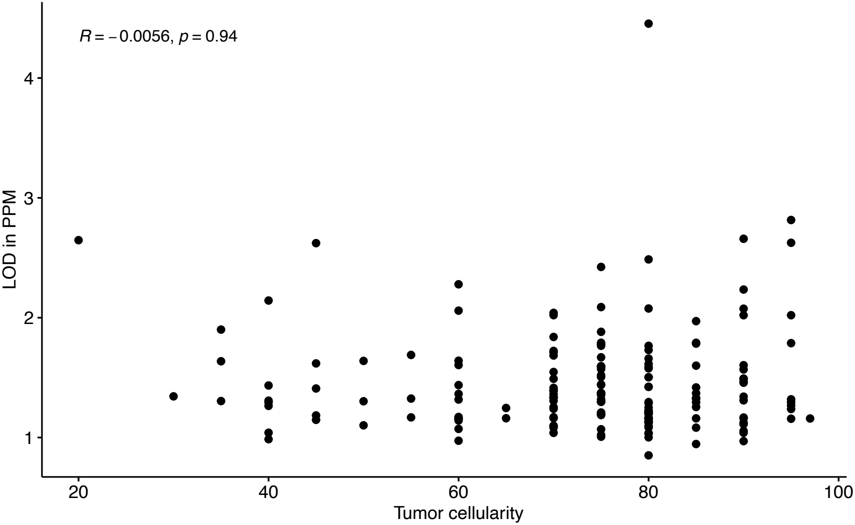

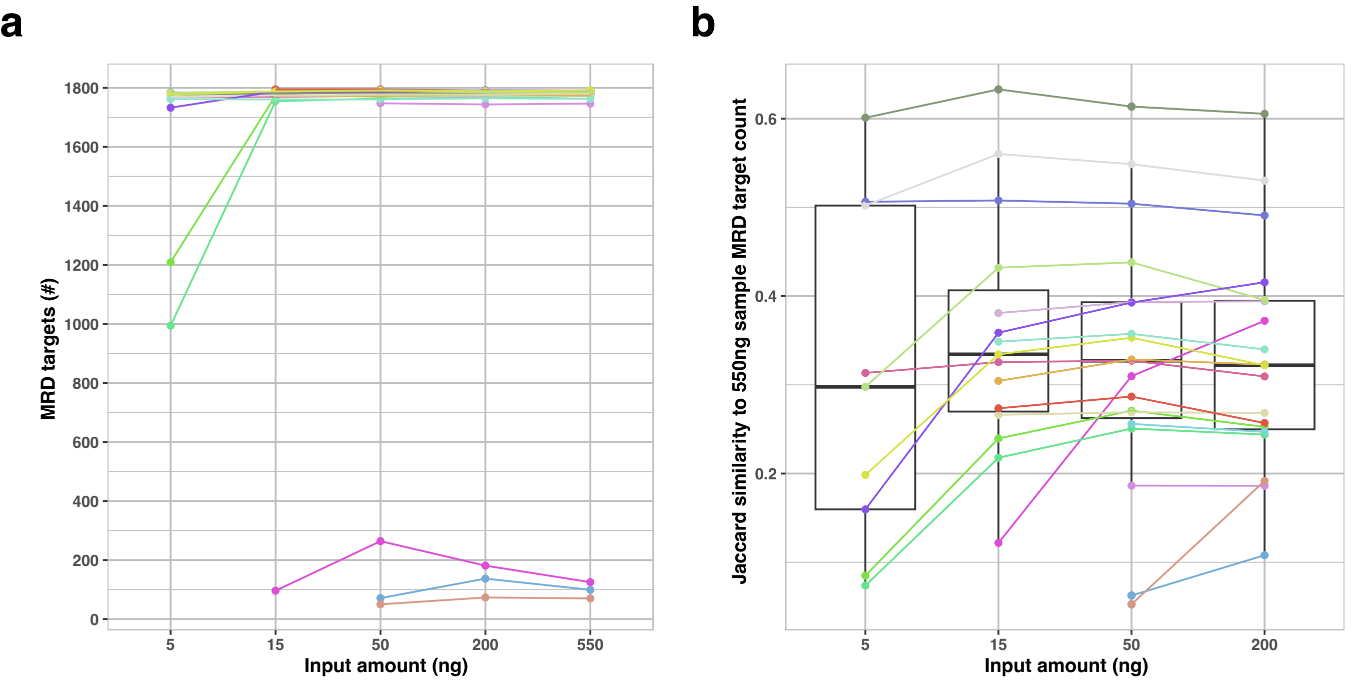

a
MRD targets (#)
1800
1600
1400
1200
1000
800
600
400
200
0
5
15
50
200
550
Input amount (ng)
b
Jaccard similarity to 550ng sample MRD target count
0.6
0.4
0.2
5
15
50
200
Input amount (ng)

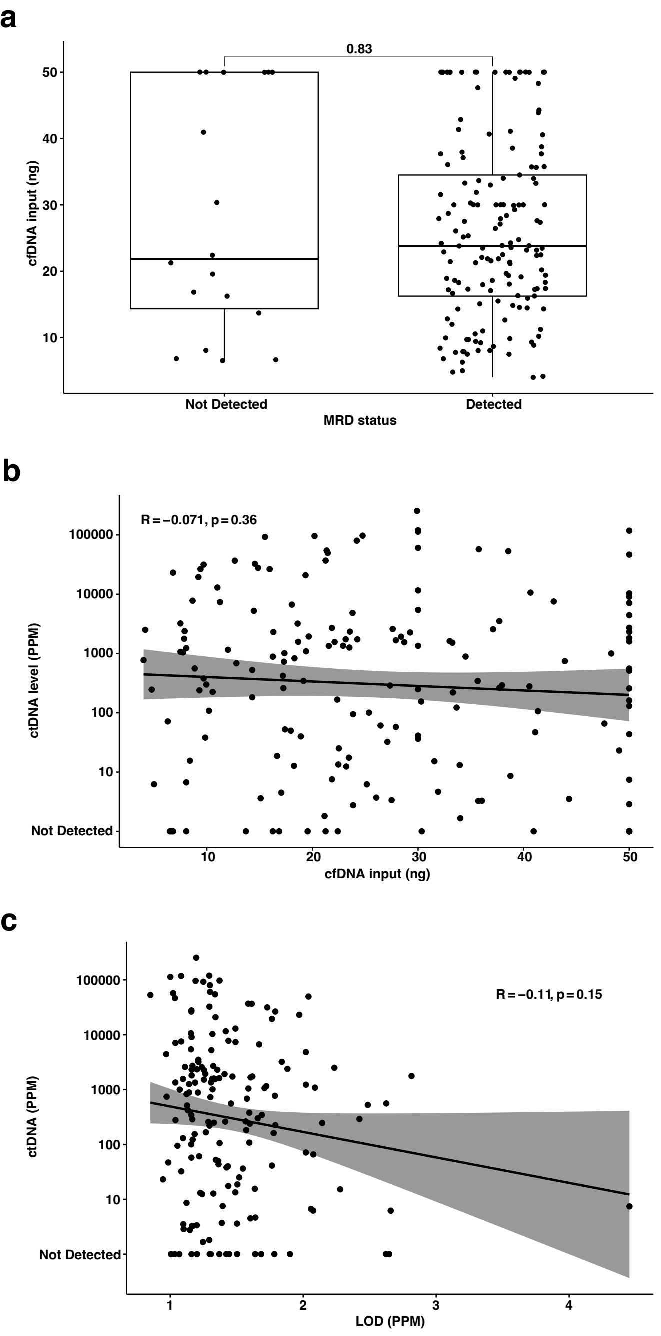

a
0.83
cfDNA input (ng)
50
40
30
20
10
Not Detected
Detected
MRD status
b
R = −0.071, p = 0.36
ctDNA level (PPM)
100000
10000
1000
100
10
Not Detected
10
20
30
40
50
cfDNA input (ng)
c
R = −0.11, p = 0.15
ctDNA (PPM)
100000
10000
1000
100
10
Not Detected
1
2
3
4
LOD (PPM)

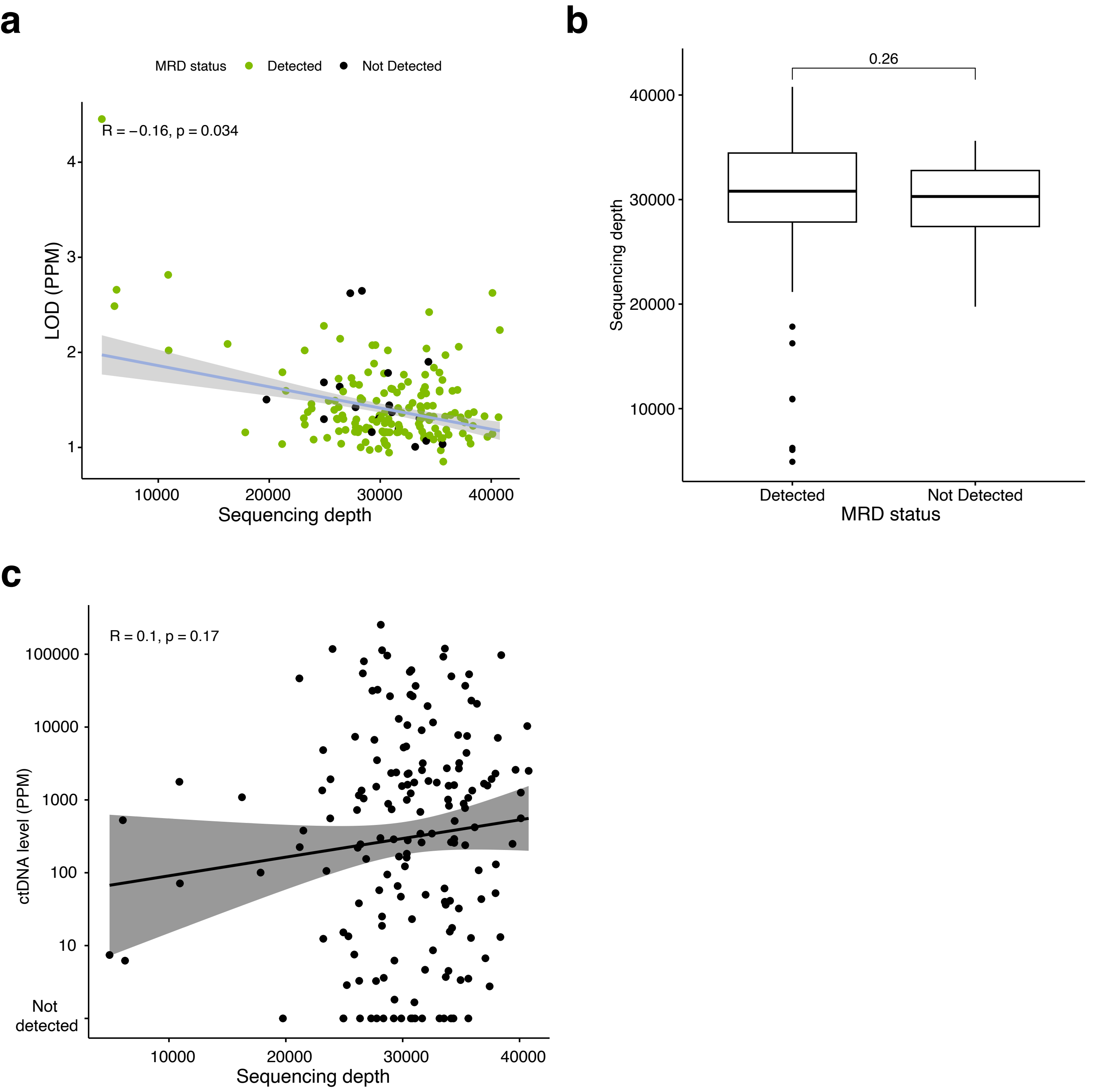
a
MRD status
Detected
Not Detected
R = −0.16, p = 0.034
LOD (PPM)
Sequencing depth
b
0.26
Sequencing depth
Detected
Not Detected
MRD status
c
R = 0.1, p = 0.17
ctDNA level (PPM)
Not detected
Sequencing depth

a

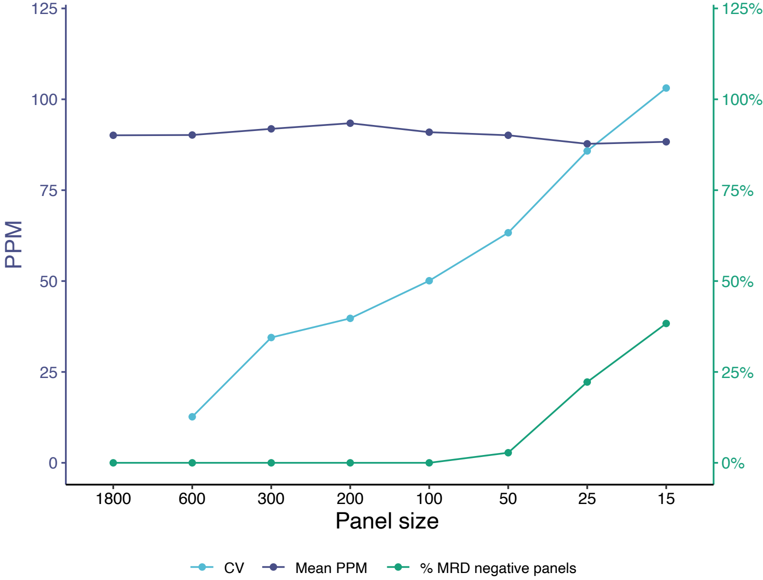
PPM
125
100
75
50
25
0
125%
100%
75%
50%
25%
0%
1800
600
300
200
100
50
25
15
Panel size
CV
Mean PPM
% MRD negative panels

Reagent	Company Name, Location	Vendor Product Number
AMPure XP beads	Beckman Coulter, Indianapolis, IN, USA	A63882
Qubit dsDNA BR	Thermo Fisher Scientific, Fremont, CA, USA	Q32850
Cell-free DNA ScreenTape	Agilent Technologies, Santa Clara, CA, USA	5067-5630
Cell-free DNA Reagents	Agilent Technologies, Santa Clara, CA, USA	5067-5631
AllPrep DNA/RNA FFPE Tissue Kit	Qiagen, Germantown, MD, USA	80234
QIAamp DNA Mini Kit	Qiagen, Germantown, MD, USA	51304
KAPA HyperPrep Kit	Roche Sequencing Solutions, Pleasanton, CA, USA	KK8504
Personalis Custom Fast Hybridization Kit, 96 rxn	Twist Bioscience, South San Francisco, CA, USA	105217
Twist Fast Wash Buffers, 96 rxn	Twist Bioscience, South San Francisco, CA, USA	100972
High Sensitivity D1000 ScreenTape	Agilent Technologies, Santa Clara, CA, USA	5067-5584
High Sensitivity D1000 Reagents	Agilent Technologies, Santa Clara, CA, USA	5067-5585
KAPA Library Quantification Kit	Roche Sequencing Solutions, Pleasanton, CA, USA	KK4824
NovaSeq 6000 instrument reagent kits	Illumina, San Diego, CA, USA	20028312
Seraseq ctDNA MRD Panel Mix	SeraCare, Gaithersburg, MD, USA	0710-2146
K2-EDTA tubes, 10 mL	BD, Franklin Lakes, NJ, USA	366643
QIAamp Circulating Nucleic Acid Kit	Qiagen, Germantown, MD, USA	55114
QIAsymphony DSP Circulating DNA Kit	Qiagen, Germantown, MD, USA	937556
