# Supplementary material for: Ultrasensitive ctDNA detection for preoperative disease stratification in early-stage lung adenocarcinoma
Source: Nat Med. 2025 Jan 13;31(1):70–6. doi: 10.1038/s41591-024-03216-y (PMC11750713; doi:10.1038/s41591-024-03216-y)
Supplement: Supplementary file 2 — Reporting Summary [file 41591_2024_3216_MOESM2_ESM.pdf]

nature portfolio

Corresponding author(s): Charles Swanton
Richard Chen
James Black

Last updated by author(s): May 16, 2024

Reporting Summary

Nature Portfolio wishes to improve the reproducibility of the work that we publish. This form provides structure for consistency and transparency in reporting. For further information on Nature Portfolio policies, see our Editorial Policies and the Editorial Policy Checklist.

Statistics

For all statistical analyses, confirm that the following items are present in the figure legend, table legend, main text, or Methods section.

n/a	Confirmed	
☐	☒	The exact sample size (*n*) for each experimental group/condition, given as a discrete number and unit of measurement
☐	☒	A statement on whether measurements were taken from distinct samples or whether the same sample was measured repeatedly
☐	☒	The statistical test(s) used AND whether they are one- or two-sided<br>*Only common tests should be described solely by name; describe more complex techniques in the Methods section.*
☐	☒	A description of all covariates tested
☐	☒	A description of any assumptions or corrections, such as tests of normality and adjustment for multiple comparisons
☐	☒	A full description of the statistical parameters including central tendency (e.g. means) or other basic estimates (e.g. regression coefficient) AND variation (e.g. standard deviation) or associated estimates of uncertainty (e.g. confidence intervals)
☐	☒	For null hypothesis testing, the test statistic (e.g. *F*, *t*, *r*) with confidence intervals, effect sizes, degrees of freedom and *P* value noted<br>*Give P values as exact values whenever suitable.*
☒	☐	For Bayesian analysis, information on the choice of priors and Markov chain Monte Carlo settings
☐	☒	For hierarchical and complex designs, identification of the appropriate level for tests and full reporting of outcomes
☐	☒	Estimates of effect sizes (e.g. Cohen's *d*, Pearson's *r*), indicating how they were calculated

Our web collection on statistics for biologists contains articles on many of the points above.

Software and code

Policy information about availability of computer code

Data collection	No software was used to collect data.
Data analysis	Personalis NeXT Personal Platform (v1.8)<br><br>R version 4.1.3<br><br>R packages:<br>tidyverse (version 1.3.2)<br>lubridate (version 1.9.2)<br>ComplexHeatmap (version 2.15.4)<br>ggplot2 (version 3.4.2)<br>ggpubr (version 0.4.0)<br>scales (version 1.2.1)<br>wesanderson (version 0.3.6)<br>ggnewscale (version 0.4.9)<br>survival (version 3.3-1)<br>survminer (version 0.4.9)<br>finalfit (version 1.0.4)<br>gt (version 0.10.1)<br>mcr (version 1.2.2)

All code needed to reproduce figures will be available on request.

For manuscripts utilizing custom algorithms or software that are central to the research but not yet described in published literature, software must be made available to editors and reviewers. We strongly encourage code deposition in a community repository (e.g. GitHub). See the Nature Portfolio guidelines for submitting code & software for further information.

Data

Policy information about availability of data

All manuscripts must include a data availability statement. This statement should provide the following information, where applicable:

- Accession codes, unique identifiers, or web links for publicly available datasets
- A description of any restrictions on data availability
- For clinical datasets or third party data, please ensure that the statement adheres to our policy

Processed TRACERx patient data has been deposited at Zenodo at the link: 10.5281/zenodo.10689003. Raw data from TRACERx patients analyzed in this study including fastq and bam files from tumour and normal WGS, as well as fastq files from cfDNA have been deposited at the European Genome–phenome Archive (EGA), hosted by The European Bioinformatics Institute (EBI) and the Centre for Genomic Regulation (CRG) under accession codes (EGAS00001006494) under controlled access.

Research involving human participants, their data, or biological material

Policy information about studies with human participants or human data. See also policy information about sex, gender (identity/presentation), and sexual orientation and race, ethnicity and racism.

Reporting on sex and gender

Sex information has been included in the data shared online. Information on patients' gender was not collected as part of the study.

Reporting on race, ethnicity, or other socially relevant groupings

Information on race, ethicity or other socially relevant groupings was not included in the manuscript.

Population characteristics

Cohort demographics (n =171) are included in Extended Data Table 1, and further patient-level details are available with supporting molecular data on Zenodo (link: 10.5281/zenodo.10689003). Inclusion and exclusion criteria for the TRACERx study (Clinical trial number: NCT01888601) are as follows:
Inclusion Criteria:
Written Informed consent
Patients ≥18 years of age, with early stage IIA-IIIB disease (according to TNM 8th edition) who are eligible for primary surgery.
Patients with a radiological staging of IB (N0) who could be upstaged to IA-IIIB following surgery (due to the presence of possible nodal involvement on the pre-operative scan) may also be included, but will be withdrawn if post-surgical staging remains IB.
Histopathologically confirmed NSCLC, or a strong suspicion of cancer on lung imaging necessitating surgery (e.g. diagnosis determined from frozen section in theatre)
Primary surgery in keeping with NICE guidelines planned (see section 9.3)
Agreement to be followed up at a TRACERx site
Performance status 0 or 1
Minimum tumour diameter at least 15mm to allow for sampling of at least two tumour regions (if 15mm, a high likelihood of nodal involvement on pre-operative imaging required to meet eligibility according to stage, i.e. T1N1-3)
Exclusion Criteria:
Any other* malignancy diagnosed or relapsed at any time, which is currently being treated (including by hormonal therapy).
Any other* current malignancy or malignancy diagnosed or relapsed within the past 3 years**.
*Exceptions are: non-melanomatous skin cancer, stage 0 melanoma in situ, and in situ cervical cancer
**An exception will be made for malignancies diagnosed or relapsed more than 2, but less than 3, years ago only if a pre-operative biopsy of the lung lesion has confirmed a diagnosis of NSCLC.
Psychological condition that would preclude informed consent
Treatment with neo-adjuvant therapy for current lung malignancy deemed necessary
Post-surgery staging is not IIA-IIIB
Known Human Immunodeficiency Virus (HIV), Hepatitis B Virus (HBV), Hepatitis C Virus (HCV) or syphilis infection.
Sufficient tissue, i.e. a minimum of two tumour regions, is unlikely to be obtained for the study based on pre-operative imaging

Recruitment

When patients are initially diagnosed with stage I-III lung cancer and then referred for surgical resection, a research nurse identifies them on a clinic/operating list. The patient has an initial eligibility assessment and is then provided with written information about the TRACERx study and he/she can ask the research nurse any questions.

Patients have to agree to provide serial blood samples whenever they attend clinic for routine blood sampling, so this represents the only main potential self-selecting bias (i.e. only patients willing to do this would participate). However, it is unclear how this would affect the biomarker analyses. Also, the gender and ethnicity characteristics are in line with patients seen in routine practice.

Ethics oversight

This study was approved by the NRES Committee London with the following details:
Study title: TRAcking non small cell lung Cancer Evolution through therapy (Rx)

REC reference: 13/LO/1546
Protocol number: UCL/12/0279
IRAS project ID: 138871
Written informed consent was obtained from all participants.

Note that full information on the approval of the study protocol must also be provided in the manuscript.

Field-specific reporting

Please select the one below that is the best fit for your research. If you are not sure, read the appropriate sections before making your selection.

☒ Life sciences ☐ Behavioural & social sciences ☐ Ecological, evolutionary & environmental sciences

For a reference copy of the document with all sections, see nature.com/documents/nr-reporting-summary-flat.pdf

Life sciences study design

All studies must disclose on these points even when the disclosure is negative.

Sample size	Here, we report analyses from 204 TRACERx patients. No sample size calculations were performed for the preoperative ctDNA substudy; sample size was determined by sample availability, and includes 18 patients from the Abbosh et al., 2017 study and 43 patients from the Abbosh et al., 2023 study. Tumor / normal WGS for 171 patients yielded passing ctDNA panels that were included for further analysis. 171 patients had preoperative plasma available, including 89 patients who had disease relapse.
Data exclusions	Patients were excluded if they did not have baseline plasma. The available plasma collected at or closest to surgery was included in the analyses for patients with multiple pre-surgical plasma. We obtained FFPE tissue for 204 patients. Of these, 62 had atypically low counts of high quality panel targets (<1,000) likely due to age and/or poor quality of the FFPE samples, and 2 did not pass panel design. For 31 of these 64 patients, DNA extracted from fresh frozen tissue was available and the remaining 33 patients were excluded from analysis.
Replication	In-silico and technical replicates were used for analytical validation of the NeXT Personal Platform. Supporting experiments are described in both the main body of the manuscript as well as in the methods, and are visualized in Figure 1 and Extended Data Figure 1.
Randomization	No randomization was performed in the this study as no therapeutic interventions are tested.
Blinding	Retrospective ctDNA analysis was conducted using prospectively collected specimens and clinical follow up. Personalis investigators were fully blinded to patient clinical outcome and clinical pathological characteristics during sample processing and ctDNA analysis. Likewise, TRACERx investigators were blinded to patient ctDNA status during clinical data and patient specimen collection.

Reporting for specific materials, systems and methods

We require information from authors about some types of materials, experimental systems and methods used in many studies. Here, indicate whether each material, system or method listed is relevant to your study. If you are not sure if a list item applies to your research, read the appropriate section before selecting a response.

Materials & experimental systems

n/a	Involved in the study	
☒	☐	Antibodies
☐	☒	Eukaryotic cell lines
☒	☐	Palaeontology and archaeology
☒	☐	Animals and other organisms
☐	☒	Clinical data
☒	☐	Dual use research of concern
☒	☐	Plants

Methods

n/a	Involved in the study	
☒	☐	ChIP-seq
☒	☐	Flow cytometry
☒	☐	MRI-based neuroimaging

Eukaryotic cell lines

Policy information about cell lines and Sex and Gender in Research

Cell line source(s)	NA
Authentication	As provided by ATCC (STR profiling).
Mycoplasma contamination	Examined by ATCC and confirmed as not detected.
Commonly misidentified lines (See ICLAC register)	None of these are commonly misidentified cell lines as reported by the ICLAC register.

Clinical data

Policy information about clinical studies

All manuscripts should comply with the ICMJE guidelines for publication of clinical research and a completed CONSORT checklist must be included with all submissions.

Clinical trial registration	TRAcking Non-small Cell Lung Cancer Evolution Through Therapy (Rx) (TRACERx): https://clinicaltrials.gov/study/NCT01888601
Study protocol	https://clinicaltrials.gov/study/NCT01888601
Data collection	Clinical and pathological data is collected from patients during study follow up - this period is a minimum of five years. Data collection is overseen by the sponsor of the study (Cancer Research UK & UCL Cancer Trials Centre) and takes place in hospitals across the United Kingdom. A centralised database called MACRO is used for this purpose. Recruitment to TRACERx started in April 2014 and is ongoing.

Outcomes

The pre-defined clinical outcome analysed in this manuscript is overall survival (OS) measured from the time of study registration to date of death from any cause. This outcome was previously defined in the TRACERx protocol (described in Jamal-Hanjani et al., 2017 NEJM). Additional analyis of relapse-free survival is also included in this manuscript, and is defined as the days from registration to any disease recurrence or new primary tumor events. The primary and secondary outcomes were pre-defined based on the following primary and secondary study objectives:

Primary objectives:
Define the relationship between intratumour heterogeneity and clinical outcome (disease-free survival and overall survival) following surgery and adjuvant therapy (including relationships between intratumour heterogeneity and clinical disease stage and histological subtypes of NSCLC).

Establish the impact of adjuvant platinum-containing regimens upon intratumour heterogeneity in relapsed disease compared to primary resected tumour.

Secondary objectives:
Development and validation of an intratumour heterogeneity ratio index as a prognostic or predictive biomarker in relation to its association with DFS and OS

Infer a complete picture of NSCLC evolutionary dynamics: Define drivers of genomic instability, metastatic progression and drug resistance by identifying and tracking the dynamics of somatic mutational heterogeneity, and chromosomal structural and numerical instability present in the primary tumour and at metastatic sites. Individual tumour phylogenetic tree analysis will:

a) Establish the order of somatic events in relation to genomic instability onset and metastatic progression

b) Decipher genetic “bottlenecking” events following metastasis and drug therapy

c) Establish dynamics of tumour evolution during the disease course from early to late stage NSCLC.

Initiate a longitudinal minimally-invasive circulating tumour cells (CTC) and cfDNA biobank to develop analytical methods for the early detection and monitoring of tumour evolution over time.

Develop a longitudinal tissue resource to serve as a platform to assess the relationship between genetic intratumour heterogeneity and the host immune response.

Isolate monocytes and lymphocytes for the in vitro generation of neoantigen-reactive T cells to be tested for immunoreactivity to matched tumour samples

Develop a repository of lung cancer cell lines, organoids and in vivo mouse models of patient- derived lung cancers that can be used as in vitro and in vivo models to study the aetiology of lung diseases, including lung cancer and predict response to therapeutics and resistance in lung cancer.

Define relationships between intratumour heterogeneity and targeted/cytotoxic therapeutic outcome.

Through UCL-GCLP gene panel in a certified laboratory environment, TRACERx will define clonally dominant disease drivers (paired primary-metastatic site comparisons in at least 270 patients with relapsed disease) to address the role of clonal driver dominance in targeted therapeutic response, and to guide stratification of lung cancer treatment and future clinical study inclusion in collaboration with the CRUK Stratified Medicines Phase II program.

Develop analytical methods for determining morphological heterogeneity within separate tumour regions.

Tissue samples obtained from lung resections, both tumour and normal, will be analysed using high-power microscopy, such as electron microscopy, in order to obtain information regarding cellular structures.

The work presented in this manuscript assessed the impact of ctDNA on patient survival via Cox regression analysis and the Kaplan Meier method.
